# The Relationship Between Population-Level SARS-CoV-2 Cycle Threshold Values and Trend of COVID-19 Infection: Longitudinal Study

**DOI:** 10.2196/36424

**Published:** 2022-11-08

**Authors:** Paria Dehesh, Hamid Reza Baradaran, Babak Eshrati, Seyed Abbas Motevalian, Masoud Salehi, Tahereh Donyavi

**Affiliations:** 1 Department of Epidemiology School of Public Health Iran University of Medical Sciences Tehran Iran; 2 Ageing Clinical and Experimental Research Team Institute of Applied Health Sciences University of Aberdeen Aberdeen United Kingdom; 3 Preventive Medicine and Public Health Research Center Tehran Iran; 4 Department of Biostatistics School of Public Health Iran University of Medical Sciences Tehran Iran; 5 Department of Biotechnology School of Allied Medical Sciences Iran University of Medical Sciences Tehran Iran

**Keywords:** cycle threshold value, COVID-19, trend, surveillance, epidemiology, disease surveillance, surveillance, digital surveillance, prediction model, epidemic modeling, health system, infectious disease

## Abstract

**Background:**

The distribution of population-level real-time reverse transcription-polymerase chain reaction (RT-PCR) cycle threshold (Ct) values as a proxy of viral load may be a useful indicator for predicting COVID-19 dynamics.

**Objective:**

The aim of this study was to determine the relationship between the daily trend of average Ct values and COVID-19 dynamics, calculated as the daily number of hospitalized patients with COVID-19, daily number of new positive tests, daily number of COVID-19 deaths, and number of hospitalized patients with COVID-19 by age. We further sought to determine the lag between these data series.

**Methods:**

The samples included in this study were collected from March 21, 2021, to December 1, 2021. Daily Ct values of all patients who were referred to the Molecular Diagnostic Laboratory of Iran University of Medical Sciences in Tehran, Iran, for RT-PCR tests were recorded. The daily number of positive tests and the number of hospitalized patients by age group were extracted from the COVID-19 patient information registration system in Tehran province, Iran. An autoregressive integrated moving average (ARIMA) model was constructed for the time series of variables. Cross-correlation analysis was then performed to determine the best lag and correlations between the average daily Ct value and other COVID-19 dynamics–related variables. Finally, the best-selected lag of Ct identified through cross-correlation was incorporated as a covariate into the autoregressive integrated moving average with exogenous variables (ARIMAX) model to calculate the coefficients.

**Results:**

Daily average Ct values showed a significant negative correlation (23-day time delay) with the daily number of newly hospitalized patients (*P*=.02), 30-day time delay with the daily number of new positive tests (*P*=.02), and daily number of COVID-19 deaths (*P*=.02). The daily average Ct value with a 30-day delay could impact the daily number of positive tests for COVID-19 (β=–16.87, *P*<.001) and the daily number of deaths from COVID-19 (β=–1.52, *P*=.03). There was a significant association between Ct lag (23 days) and the number of COVID-19 hospitalizations (β=–24.12, *P*=.005). Cross-correlation analysis showed significant time delays in the average Ct values and daily hospitalized patients between 18-59 years (23-day time delay, *P*=.02) and in patients over 60 years old (23-day time delay, *P*<.001). No statistically significant relation was detected in the number of daily hospitalized patients under 5 years old (9-day time delay, *P*=.27) and aged 5-17 years (13-day time delay, *P*=.39).

**Conclusions:**

It is important for surveillance of COVID-19 to find a good indicator that can predict epidemic surges in the community. Our results suggest that the average daily Ct value with a 30-day delay can predict increases in the number of positive confirmed COVID-19 cases, which may be a useful indicator for the health system.

## Introduction

Coronaviruses are zoonotic pathogens that can be transmitted to humans after acquiring particular mutations [1]. SARS-CoV-2, which causes COVID-19, is mainly transmitted via airborne respiratory droplets. Although ocular secretions and oral-fecal transmission have also been indicated, these transmission methods remain uncertain [2,3].

A real-time reverse transcription-polymerase chain reaction (RT-PCR) test is used for detecting SARS-CoV-2 in respiratory samples as routine surveillance worldwide. The RT-PCR test has high sensitivity and specificity for diagnosing COVID-19 and offers faster turnaround times than the viral culture method; thus, this test has become the main method for diagnosing COVID-19. RT-PCR presents both qualitative and quantitative results with respect to the viral load [4]. The RT-PCR cycle threshold (Ct) value is identified as the number of amplification cycles needed to detect the target gene in samples [5]. The Ct value is a semiquantitative result of RT-PCR that reflects the amount of viral nucleic acids in a sample, and can thus be used as a proxy for viral load and may help decision-making in epidemic control. The Ct value has a reverse relationship with viral load so that each 3.3 increase in Ct value causes a 10-fold decrease in viral load [6]; the highest viral burden is on the first day of disease symptoms onset [7]. The positive result of COVID-19 RT-PCR tests has a lower Ct value than the recommended cutoff. In the United States, the Food and Drug Administration considers a Ct value <37 as the cutoff for a positive result of COVID-19 [8]. In more than 70% of samples with a Ct value <25, SARS-CoV-2 may be cultured, whereas only 3% of samples with a Ct value >35 can be cultured [9]. Several studies have reported that the Ct value also has an association with disease severity and mortality, and that the Ct values in patients who have more severe symptoms are low [5,10-12]. In addition, hospitalized patients who died from COVID-19 had lower Ct values [13]. A systematic review showed a significant correlation between Ct value and disease severity in hospitalized patients but not in nonhospitalized COVID-19 patients [5]. There is controversy among studies on the use of Ct values at an individual level for the prognosis of the disease or treatment planning. The Ct value may vary due to the collection method among laboratories [14] or the target gene selected for RT-PCR [15]. Moreover, the RT-PCR test can detect any viral material and does not distinguish between live viruses and viral debris, which may persist for a long time beyond the point of infectiousness [12].

To the best of our knowledge, few studies have examined the use of population-level Ct values as a measure of COVID-19 dynamics in communities. As Ct values have a significant relationship with disease severity and infectivity, a higher average Ct value in daily testing samples from a population may predict epidemic growth in a community. Hay et al [16] analyzed simulation and surveillance data and found that decreases in the proportion of Ct values in a population may cause a local increase in transmission or a new number of patients [16]. In addition, the median Ct value may be an effective measure for forecasting a pandemic surge.

To resolve these issues, the aims of this study were to determine the relationships between the daily trend of average Ct value and COVID-19 dynamics, including the daily number of hospitalized patients with COVID-19, daily number of new positive tests, daily number of COVID-19 deaths, and number of hospitalized patients with COVID-19 by age. We further aimed to determine the lag between these series.

## Methods

### Samples and RT-PCR

The samples included in this study were collected from March 21, 2021, to December 1, 2021. Inclusion criteria were samples obtained from individuals suspected of having COVID-19 and were referred to a laboratory in Tehran, Iran, to confirm the diagnosis. Daily results of Ct values of all patients referred to the laboratory for RT-PCR tests were recorded. The daily number of positive cases and the number of hospitalized people by age group for 9 months were extracted from the COVID-19 patient information registration system in Tehran province, Iran.

This study included samples of the upper respiratory tract (both nasopharyngeal and anterior nares swab samples) taken using a sterile Dacron thin swab with a plastic or aluminum handle as the main test specimen. The samples were collected by a physician, nurse, laboratory expert, and other staff with sufficient training and experience. All biological samples were sent to the Molecular Diagnostic Laboratory of Iran University of Medical Sciences in Tehran, Iran. All samples were analyzed using the Pishtazteb One-step RT-PCR COVID-19 Kit (dual-target gene diagnosis), and RNA extraction was performed using a Zybio nucleic acid extraction kit (magnetic bead method). To confirm the diagnosis, the target genes were the SARS-CoV-2 nucleocapsid gene and RdRp gene [17]. For each sample, the Ct value was recorded. The samples that produced a positive result in the RT-PCR test and had a Ct value ≤37 were recorded to determine the daily average Ct values.

### Statistical Analysis

#### Overview

The daily median Ct value among all patients referred to the laboratory and the daily number of hospitalized patients with COVID-19 by age group were plotted over time. The autoregressive integrated moving average (ARIMA) and autoregressive integrated moving average with exogenous variables (ARIMAX) models were used to determine significant associations between the daily average Ct value and the daily number of COVID-19 hospitalizations by age, daily number of COVID-19 deaths, and daily number of positive tests in Tehran province, Iran.

#### ARIMA Model

Time-series analyses are appropriate when dealing with a set of data that has a time trend [18]. The Box-Jenkins time-series approach, especially the ARIMA model, is one of the best methods in time-series analysis of autocorrelated data [19], such as the daily average Ct value. In autoregressive models, the outcome (Y_t_) is a linear function of the previous values and a random component. Nonseasonal ARIMA model parameters are (*p, d, q*) overall, where *p* is the order of autoregression (AR), *d* is the degree of trend difference, and *q* is the order of moving average (MA). To perform time-series analysis, it is first necessary to check the stability of the mean and variance. For this purpose, the augmented Dickey-Fuller (ADF) test is used [20] for checking the stability of the mean and the Box-Cox test is used to check the stability of the variance. Logarithm transformation and differentiation were used to establish stability in the variance and mean, respectively. The first-time differences can be expressed as:

Y′_t_=Y_t_–Y_t–1_ **(1)**

Where Y_t_ represents nonstationary time-series data and Y′_t_ is the time series after the first-time differences. If the time series has a seasonal trend, seasonal differences are used to stabilize the series. The AR parameter *p* represents the linear correlation of the current value of the time series Y_t_ with the previous values Y_t–1_, Y_t–2_,... and current residuals ε_t_ [21]. The MA parameter *q* shows the linear correlation of the current value of the time series Y_t_ with the current and previous residuals of the time series ε_t_, ε_t–1_,… [22]. The general formula of AR (*p*) and MA (*q*) models are represented in equations (2) and (3), respectively:

Yt=C+β_1_Y_t–1_+β_2_Y_t-2_+…+βpYt–n+ε_t_  **(2)**

Yt=C+ε_t_–ϕ_1_ε_t–1_–ϕ_2_ε_t–2_…–ϕ_q_ε_t–q_   **(3)**

where C is a constant; β_1_, β_2_,…, β_p_ are AR model terms; and ϕ_1_, ϕ_2_,…, ϕ_q_ are MA model terms. The number of AR and MA parameters was determined by the autocorrelation function and partial autocorrelation function.

The general form of the ARIMA model can be written as:

Y′_t_=C+β_1_Y_t–1_+β_2_Y_t–2_+…+β_p_Y_t–p_+ϕ_1_ε_t–1_+ϕ_2_ε_t–2_+…+ϕ_q_ε_t–q_+ε_t_… **(4)**

Four main steps for the development of the ARIMA model include checking mean and variance stability (see Table S1 in Multimedia Appendix 1), and identifying *p* and *q* terms (see Figure S1 in Multimedia Appendix 1).

#### Model Parameter Estimation

The maximum-likelihood approach was used for the model parameters. To determine the best ARIMA model, among the models that passed the residual test (normality and stability in the variance), the model with the lowest Bayesian information criterion (BIC) and Akaike information criterion (AIC) was selected as the final model. The BIC and AIC formulae are represented as follows:

BIC=–2.ln(*L*)+k.ln(m)   **(5)**

AIC=2k–2ln(*L*)   **(6)**

Where m is the number of observations, k is the total number of parameters in the model, and ln(*L*) is the likelihood function.

The ARIMA model was developed to the time series of the daily average Ct value, daily number of hospitalized patients with COVID-19, new number of daily positive tests, daily number of COVID-19 deaths, and number of hospitalized patients with COVID-19. The detailed method for derivation of the ARIMA model is described in Multimedia Appendix 1.

#### Cross-correlation Function

To evaluate the time delay between the daily average Ct value and the daily number of hospitalized patients with COVID-19, daily number of new positive tests, daily number of COVID-19 deaths, and number of hospitalized patients with COVID-19 by age, the cross-correlation function was used. The independent (daily average Ct value) and dependent variables (daily number of hospitalized patients with COVID-19, new number of daily positive tests, daily number of COVID-19 deaths, and number of hospitalized patients with COVID-19 by age) were preprocessed by the previously fit ARIMA models. The cross-correlation coefficient is mathematically represented as follows:

*r_αβ_*_(_*_k_*_)_=C*αβ(k)*/*S_α_S_β_*   **(7)**

where C*αβ(k)* is the value of covariance between the preprocessed input time series and preprocessed output time series at the lag *k*, 𝑆_𝛼_ is the value of the standard deviation of the preprocessing input time series, and 𝑆_𝛽_ is the value of the standard deviation of the preprocessing output time series [23]. Three indicators, Schwarz Bayesian information criterion (SBIC), Hannan-Quinn information criterion (HQIC), and AIC, were used to select the best lag.

SBIC=log(n)k–2 log(L(θ̂))   **(8)**

HQIC=–2ln(L(θ̂)) +2klog(logn)   **(9)**

In equations (8) and (9), n is the sample size, k is the number of estimated parameters, θ is the set of all parameter values, and L(θ̂) is the likelihood of the model.

#### ARIMAX Model

**Figure 1:**
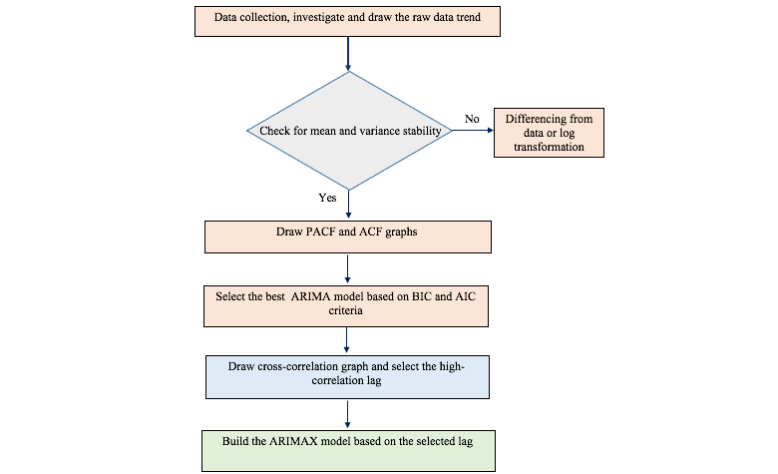
Steps of building the best ARIMAX model. ACF: autocorrelation function; AIC: Akaike information criterion; ARIMA: autoregressive integrated moving average; ARIMAX: autoregressive integrated moving average with exogenous variables; BIC: Bayesian information criterion; PACF: partial autocorrelation function.

The ARIMAX model is an expansion of the ARIMA model by adding an explanatory independent variable. The ARIMAX model is the combination of multiple regression analysis and time-series analysis; therefore, it can determine the impact factor of the relationship between different lags of Ct values and other study variables. The ARIMAX model formula is as follows:

Y_t_=βx(t)+α_1_Y_t–1_+α_2_Y_t–2_+…+α_p_Y_t–p_+ε_t_–ϕ_1_ε_t–1_+ϕ_2_ε_t–2_+…+ϕ_q_ε_t–q_+ε_t_…   **(10)**

where x(t) is an independent variable at time t and β is its associated coefficient. Y_t–1)_…Y_t−p_ is the previous value of a dependent variable, and ε_t_…ε_t–q_ is the residual of the time series. To determine the association and coefficient of the association between the lags of the x_t+m_ time series and series Y_t_, the ARIMAX model was used. The cross-correlation function was used to find the linear correlation between x_t+m_ and Y_t_ for different lags, which can help to find the best lags of the independent variable that might be used to predict the dependent variable [24]. The lags of Ct values that were selected through the correlation function were incorporated as covariates into the ARIMAX model with other dependent variables such as the daily number of hospitalized patients with COVID-19, number of new daily positive cases, daily number of COVID-19 deaths, and number of hospitalized patients with COVID-19 by age. The maximum-likelihood method was used for estimation of the parameters. The Ljung-Box Q test was applied to evaluate white noise for the residual series. Data were analyzed by Stata software version 14. Figure 1 shows the steps of building the best ARIMAX model.

### Ethics Considerations

Since individual data were not used in this study, no formal ethical assessment or informed consent was required. This study was approved by the Ethics Committee of Iran University of Medical Sciences (ethical code: IR.IUMS.REC.1400.799).

## Results

### Evaluation Outcomes

Table 1 shows descriptive statistics of the study variables that were included in the analysis. The minimum value of Ct was related to April 11, 2021, and the maximum frequency of hospitalized patients was related to August 23, 2021. Over 9 months, 80,882 positive COVID-19 tests were referred to the Molecular Diagnostic Laboratory of Iran University of Medical Sciences in Tehran, Iran.

**Figure 2:**
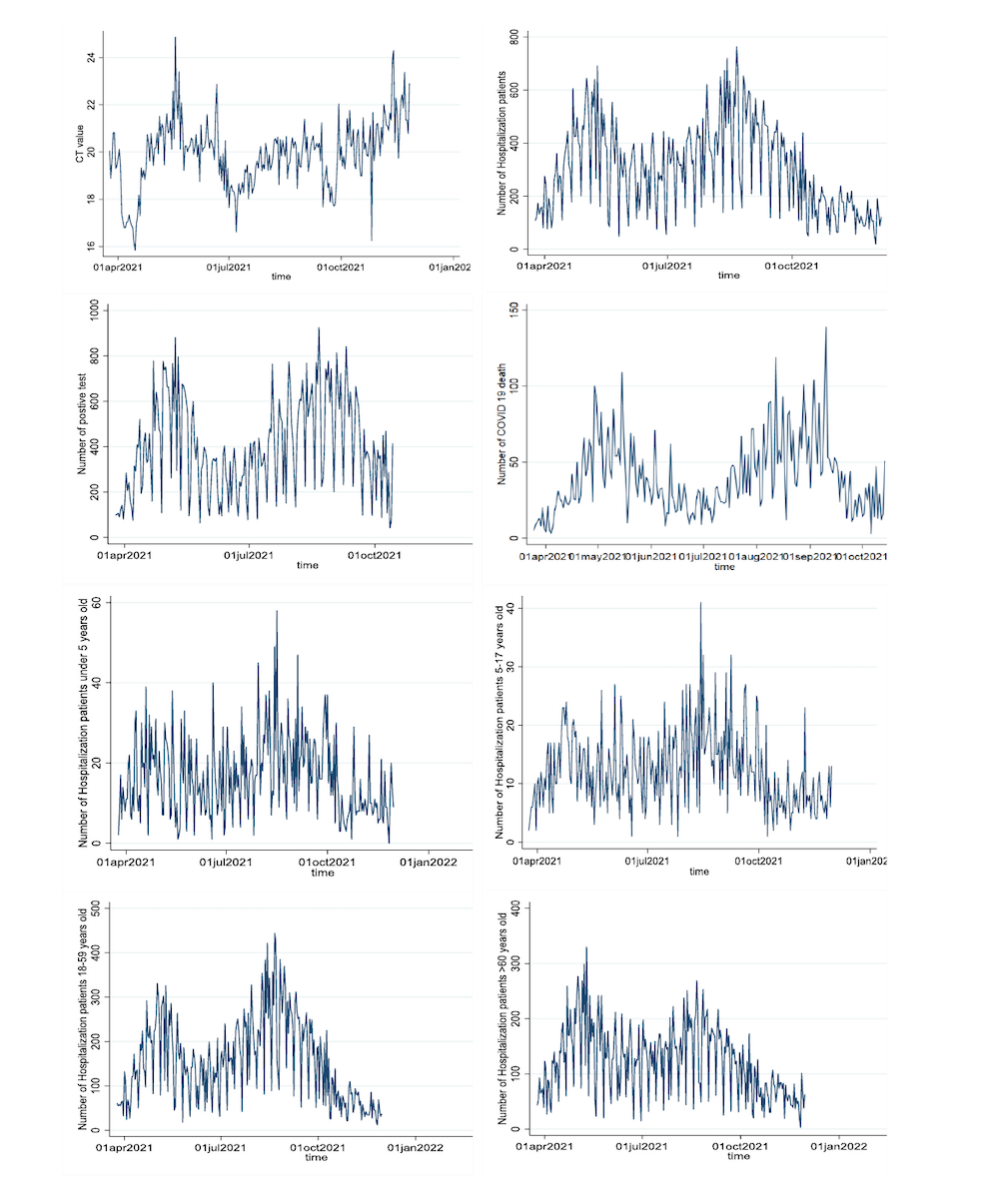
Trends of cycle threshold (Ct) values and other study variables over 9 months.

Figure 2 shows the time trend of Ct values, along with the trends of the number of hospitalized patients, number of positive tests, number of COVID-19 deaths, number of hospitalized patients under 5 years old, number of hospitalized patients aged 5-17 years old, number of hospitalized patients aged 18-59 years old, and number of hospitalized patients over 60 years old over the 9 months. Similar to the a priori hypothesis, the daily average Ct value was negatively correlated with the daily number of hospitalized patients, daily count of positive COVID-19 tests (with a time delay), daily number of COVID-19 deaths, and daily number of hospitalized patients by age group. As shown in Figure 2, there was a time delay of approximately 28-32 days between the average daily Ct value and the daily number of hospitalized patients with COVID-19, daily count of positive COVID-19 tests, and daily number of COVID-19 deaths.

**Table 1:** Descriptive statistics of the study variables.

Variables		Maximum	Minimum	Mean (SD)
Dependent variable: cycle threshold value		24.87	15.83	19.89 (1.33)
**Independent variables**				
	Number of hospitalized patients	763	47	310.65 (260.259)
	Number of positive tests	925	42	396.48 (211.05)
	Number of COVID-19 deaths	72	0	15.98 (24.57)
	Number of hospitalized patients under 5 years old	58	0	16.514 (10.23)
	Number of hospitalized patients aged 5-17 years	41	1	12.35 (6.78)
	Number of hospitalized patients aged 18-59 years	444	12	155.94 (91.61)
	Number of hospitalized patients over 60 years old	330	3	123.58 (63.37)

### ARIMA Model for Study Variables

Table 2 shows the best ARIMA models for the study variables. The ARIMA (1,0,1) model was the best model for the daily average Ct value in comparison with other models, having the lowest BIC value, daily number of the hospitalized patients, and daily count of positive COVID-19 tests. The ARIMA (1,0,2) model was the best model for the daily number of COVID-19 deaths. All models had the lowest number of significant estimated parameters, and the residual analysis showed a good fit (normality and stability in the variance) for the selected ARIMA models using the AIC. There was no seasonal pattern in the study variables. The ADF test was used for evaluating stability in the mean and the Box-Cox test was used to test the time-series stability in the variance. The time series of the daily number of hospitalized patients by age did not show stability for the variance, and therefore log transformation was applied to this variable.

**Table 2:** The best selected autoregressive integrated moving average (ARIMA) models using the Bayesian information criterion (BIC) and Akaike information criterion (AIC).

Variable	ARIMA	Log likelihood	AIC	BIC
Cycle threshold value	(1,0,1)^a^	–355.99	702.38	716.42
Number of hospitalized patients	(1,0,1)	–1553.31	–1231.24	–1220.61
Number of positive COVID-19 tests	(1,0,1)	–1198.16	2494.99	2827.07
Number of COVID-19 deaths	(1,0,2)	–933.16	1876.33	1893.88
Number of hospitalized patients under 5 years old	(1,0,1)	–905.64	481.26	494.797
Number of hospitalized patients aged 5-17 years	(1,0,1)	–819.80	393.80	407.322
Number of hospitalized patients aged 18-59 years	(1,0,1)	–1401.00	374.58	384.72
Number of hospitalized patients over 60 years old	(1,0,1)	–919.60	397.41	407.55

^a^The numbers in parentheses represent the parameters (*p*, *d*, *q*) of the model, where *p* is the order of autoregression, *d* is the degree of trend difference, and *q* is the order of moving average.

### Cross-correlation Analysis

**Figure 3:**
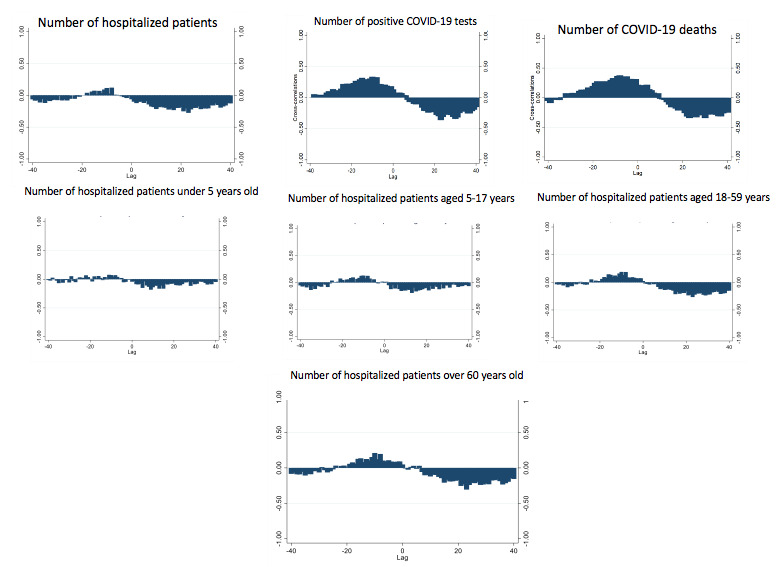
Cross-correlations (y-axes) between cycle threshold (Ct) values and other study variables.

Figure 3 shows the cross-correlations between the study variables and Ct value. In this figure, negative lags would not be considered because the negative lag indicates that the study variables could affect the average Ct value in a certain period at a later point in time; therefore, the positive lag was used to show the effect of the Ct value on the study variables in the future. A cross-correlation function was performed between the preprocessed input and output series. Table 3 shows the best lag difference between the Ct value and the study variables. Indicators such as AIC, SBIC, and HQIC were used to examine the selected lag. There was no statistically significant (all *P*>.05) lag (time delays) between the average Ct value and the daily number of hospitalized patients under 5 years old and the number of hospitalized patients aged 5-17 years. However, a significant 23-day lag was found between the average Ct value and number of hospitalized patients. The daily count of positive COVID-19 tests as well as the daily number of COVID-19 deaths had a significant 30-day lag with the average Ct value.

**Table 3:** High-correlation lags between the cycle threshold value and other study variables.

Variable	Lag	*r*	*P* value	AIC^a^	HQIC^b^	SBIC^c^
Number of hospitalized patients	23	–0.25	.02	15.34	15.95	16.85
Number of positive tests	30	–0.34	.02	15.83	16.83	8.24
Number of COVID-19 deaths	30	–0.26	.02	10.90	11.69	12.86
Number of hospitalized patients under 5 years old	9	–0.22	.76	10.38	10.73	11.25
Number of hospitalized patients aged 5-17 years	13	–0.23	.29	9.54	9.89	10.41
Number of hospitalized patients aged 18-59 years	23	–0.27	.04	14.24	14.85	15.75
Number of hospitalized patients over 60 years old	23	–0.30	.07	13.54	14.15	15.05

^a^AIC: Akaike information criterion.

^b^HQIC: Hannan-Quinn information criterion.

^c^SBIC: Schwarz Bayesian criterion.

### Impact of the Ct Value on Study Variables (ARIMAX Model)

After obtaining the best lag between the daily Ct value and other variables using cross-correlation analysis (Table 3), ARIMAX was used to calculate the impact coefficients of the selected lags. Table 4 shows that a Ct value with a 30-day delay could affect the daily number of positive COVID-19 tests and the daily number of deaths from COVID-19. Specifically, a decrease in Ct value may cause an increase of approximately 16.87 times in the average number of new positive tests for COVID-19 after 30 days. In addition, the daily number of deaths from COVID-19 will increase by approximately 1.52 times after 30 days with a decrease in the Ct value. There was a significant coefficient between Ct lag (23 days) and the number of COVID-19 hospitalizations. There was also a significant association of the Ct value with a 23-day delay and the number of COVID-19 hospitalizations for patients aged 18-59 years and patients aged more than 60 years.

**Table 4:** Estimated coefficients obtained using autoregressive integrated moving average with exogenous variables models.

Variables and parameters			Coefficient (β)		95% CI		*P* value
**Number of hospitalized patients; best model: (1,0,1)**							
	Ct^a^ (23)^b^	–24.12		–41.08 to –7.16		.005	
	AR^c^ (1)	.99		.95 to 1.02		<.001	
	MA^d^ (1)	–.87		–.96 to –.78		<.001	
**Number of COVID-19 deaths; best model: (1,0,2)**							
	Ct (30)	–1.52		–2.86 to –.18		.03	
	AR (1)	.96		.89 to 1.03		<.001	
	MA (1)	–1.07		–1.22 to –.92		<.001	
	MA (2)	.21		.09 to .34		.001	
**Number of positive tests; best model: (1,0,1)**							
	Ct (30)	–16.87		–28.93 to –4.82		<.001	
	AR (1)	.96		.84 to 1.07		<.001	
	MA (1)	–.89		–1.06 to –.71		<.001	
**Number of hospitalized patients under 5 years old; best model: (1,0,1)**							
	Ct (9)	–.60		–1.68 to .47		.27	
	AR (1)	.96		.84 to 1.07		<.001	
	MA (1)	–.89		–1.06 to –.71		<.001	
**Number of hospitalized patients aged 5-17 years (1,0,1)**							
	Ct (13)	–.40		–1.30 to .50		.39	
	AR (1)	.97		.92 to 1.03		<.001	
	MA (1)	–.89		–.99 to –.79		<.001	
**Number of hospitalized patients aged 18-59 years; best model: (1,0,1)**							
	Ct (23)	–11.87		–21.81 to –1.94		.02	
	AR (1)	.99		.95 to 1.02		<.001	
	MA (1)	–.85		–.94 to –.76		<.001	
**Number of hospitalized patients over 60 years old; best model: (1,0,1)**							
	Ct (23)	–11.44		–17.82 to –5.07		<.001	
	AR (1)	.99		.96 to 1.02		<.001	
	MA (1)	–.90		–.98 to –.81		<.001	

^a^Ct: cycle threshold.

^b^The numbers in parentheses indicate the lag in days.

^c^AR: autoregressive.

^d^MA: moving average.

## Discussion

### Principal Findings

The Ct value is a good proxy for viral load, which can offer the possibility of isolating people who have a higher viral load (lower Ct value) and those who have been in contact with these people for the past 5 days to reduce the transmission rate [11]. Therefore, the Ct value can be a good indicator for predicting the state of the disease process in the future. This study investigated the relationship between the population distribution of Ct values obtained from SARS-CoV-2–positive RT-PCR tests and COVID-19 dynamics. The results showed that the daily average Ct value has a significant negative relationship with three study variables of COVID-19 dynamics: daily number of hospitalized patients, daily count of positive COVID-19 tests, and daily COVID-19 deaths. The Ct value can predict the peak of the epidemic curve of the number of new positive COVID-19 patients with an interval of 30 days earlier.

### Comparison With Prior Work

This result is consistent with the results of a study by Walker et al [21] showing that a declining population-level Ct value preceded increases in SARS-CoV-2 positivity tests. Another study showed a negative association between individual Ct values and severity of symptoms of COVID-19 [25]. A few studies have focused on the effect of the population-level Ct value as an indicator for predicting pandemic surges. Consistent with this study, Tso et al [26] showed that daily median Ct values have a negative correlation with the daily count of positive tests, daily transmission rates, and daily number of COVID-19 hospitalizations in the greater El Paso area; they also showed a significant 33-day time delay between daily median Ct values and the daily number of COVID-19 hospitalizations. In this study, we found a significant 23-day time delay between the daily average Ct value and the number of hospitalized COVID-19 patients aged 18-59 years and aged more than 60 years. The former age group represents the major workforce, and are thus more likely to be exposed and become infected with the SARS-CoV-2 virus. Buchan et al [27] showed that the average Ct values were statistically similar among age groups, but patients in the age group of 80-89 years had slightly lower Ct values. According to an epidemiology study in Iran, the majority of hospitalized COVID-19 patients were in the age group of 50-60 years [28]. The relationship between the daily average Ct value and the number of COVID-19 patients aged under 5 years was not significant in this study.

Hay et al [16] estimated the epidemic trajectory in Massachusetts, United States, using a mathematical model for population-level Ct values, and also found that an increasing epidemic wave will be accompanied by a high frequency of recently infected patients with high viral loads (lower Ct values), whereas a declining epidemic wave occurs when the number of patients with older infections is high. Therefore, Ct values obtained from the disease care system during the epidemic of SARS-CoV-2 can determine the course of the epidemic process at short intervals [16]. In this study, the ARIMAX model was used to find the effect of Ct value delay time on the number of positive COVID-19 tests, and a 30-day delay was found between the average population-level Ct value and the number of positive COVID-19 cases.

### Limitations

Differences in how measurements of Ct value or assurance about the quality of the data sets that are used to measure population-level Ct values in different geographical areas may affect the power of the Ct value for predicting local COVID-19 epidemic waves. Previous studies have indicated that changes in the population-level Ct values of surveillance samples may lead to a disease outbreak [16,29]. There is a hypothesis that if only patients with clinical symptoms who had positive tests were used to calculate the daily average Ct value, the association between the daily Ct value and COVID-19 cases would be more readily detected; thus, a decrease in Ct values may be more closely associated with the increasing number of COVID-19 patients. To investigate this hypothesis, only the Ct value of patients with symptoms was used to calculate the daily average Ct value in this study.

### Conclusions

The daily average population-level Ct value has a relationship with the number of positive SARS-CoV-2 tests and time delay. Thirty days after reducing the daily average Ct value, the number of new COVID-19 cases is expected to increase. It is important to find a good indicator that can predict epidemic surges in the community for improved COVID-19 surveillance. Faster prediction of a new wave of disease will help health policymakers to initiate appropriate public health policies such as lockdowns for decreasing an anticipated pandemic surge, and will provide health systems an opportunity to meet the needs of medicine and facilities to support additional patients.

## Appendix

Multimedia Appendix 1 Additional data related to the statistical analysis steps and detailed description of the ARIMA model.

## Abbreviations

ADF: augmented Dickey-Fuller
AIC: Akaike information criterion
AR: autoregressive
ARIMA: autoregressive integrated moving average
ARIMAX: autoregressive integrated moving average with exogenous variables
BIC: Bayesian information criterion
Ct: cycle threshold
HQIC: Hannan-Quinn information criterion
MA: moving average
RT-PCR: reverse transcription-polymerase chain reaction
SBIC: Schwarz Bayesian information criterion
