# Supplementary material for: The Relationship Between Population-Level SARS-CoV-2 Cycle Threshold Values and Trend of COVID-19 Infection: Longitudinal Study
Source: JMIR Public Health Surveill. 2022 Nov 8;8(11):e36424. doi: 10.2196/36424 (PMC9645421; doi:10.2196/36424)
Supplement: Multimedia Appendix 1 [file publichealth_v8i11e36424_app1.docx]

**Autoregressive integrated moving average (ARIMA) model**

Autoregressive Integrated Moving Average **(**ARIMA) models or the Box-Jenkins model is the well-known approach in the time series analysis. These models require data that has a time trend. A non-seasonal ARIMA (p,d,q as defined in the main text) model included 3 parameters; p and q are the defined as autoregressive (AR) and moving average (MA), and d defined as the number of necessary differences to stabilize the series. Logarithm transformation and differentiation were used to establish stability in these series. Parameters p and q were determined based on the partial autocorolation graph and Autorcorrolation graph. The general form of the ARIMA model can be written as

$${y'}_{t}={\alpha+\beta_{1}y'}_{t-1}+\beta_{2}{y'}_{t-2}+ \ldots+\beta_{p}{y^{'}}_{t-p}+\theta_{1}\varepsilon_{t-1}+\theta_{2}\varepsilon_{t-2}+ \ldots+\theta_{q}\varepsilon_{t-q}+\varepsilon_{t}\ldots. (1)$$

where ${y'}_{t}$ is the time series and in the right side of the model other the predictors are lagged values of $y_{t}$ and lagged errors. $\beta_{1}, \beta_{2}\ldots\beta_{p}$ are the coefficients of parameters aof p and $\theta_{1}, \theta_{2}\ldots\theta_{q}$ are the coefficients of q parameter.

The ARIMAX model is used to determine the relationship between two series and to determine the impact coefficients. An ARIMAX model can be expressed as follows:

$$y_{t}= \beta_{0}+\beta_{1}x_{1,t}+\ldots+\beta_{k}x_{k,t}+\varepsilon_{t}\ldots.(2)$$

where $y_{t}$ is equal to the time series of the response variable, $x_{1,t}\ldots x_{k,t}$ are the covariates of time series that may have assosication with$y_{t}$ and $\varepsilon_{t}$ is the error. Notice that the $\varepsilon_{t}$ in this model contine as two error; both of errors from the regression model the ARIMA model.

|  | 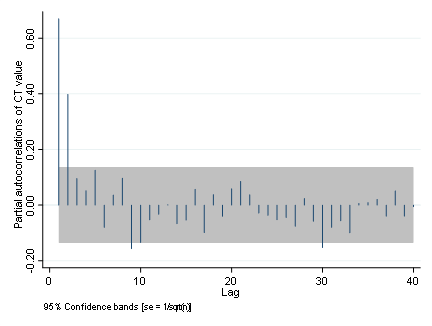 |
| --- | --- |
|  |  |
|  |  |
|  |  |
|  |  |
|  |  |
|  |  |
|  |  |

**Figure S1** Partial autocorrelation graph of study variables.

| **Table S1** The Dickey–Fuller Test of the study variables | | |  |
| --- | --- | --- | --- |
| **variables** | | **Original series** | |
|  |  | Z-Statistic | P-value |
| CT value | | -7.064 | 0.0001 |
| Number of Hospitalization patients | | -9.144 | 0.0001 |
| Number of postive test | |  |  |
| Number of COVID 19 death | | -12.415 | 0.0001 |
| Number of Hospitalization patients under 5 years old | | -12.678 | 0.0001 |
| Number of Hospitalization patients 5-17 years old | | -13.74 | 0.0001 |
| Number of Hospitalization patients 18-59 years old | | -8.279 | 0.0001 |
| Number of Hospitalization patients >60 years old | | -10.38 | 0.001 |

| **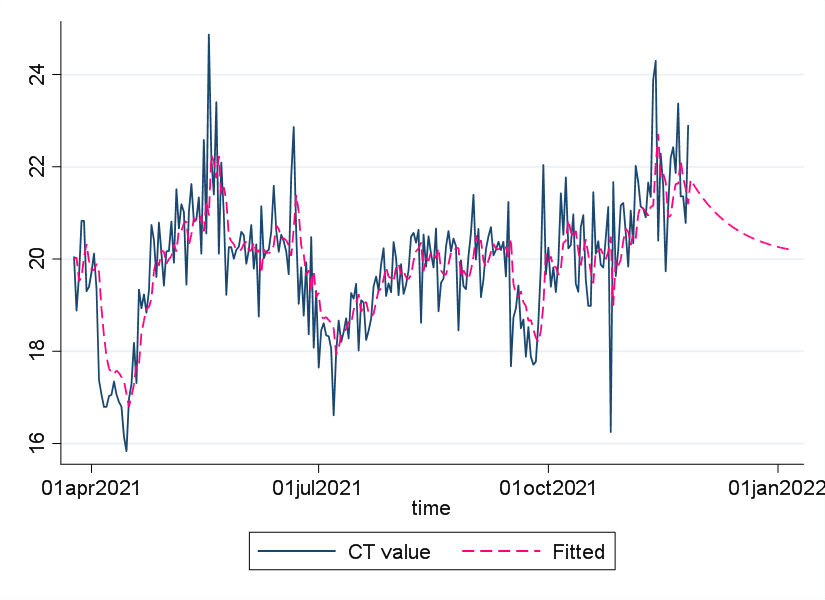** | **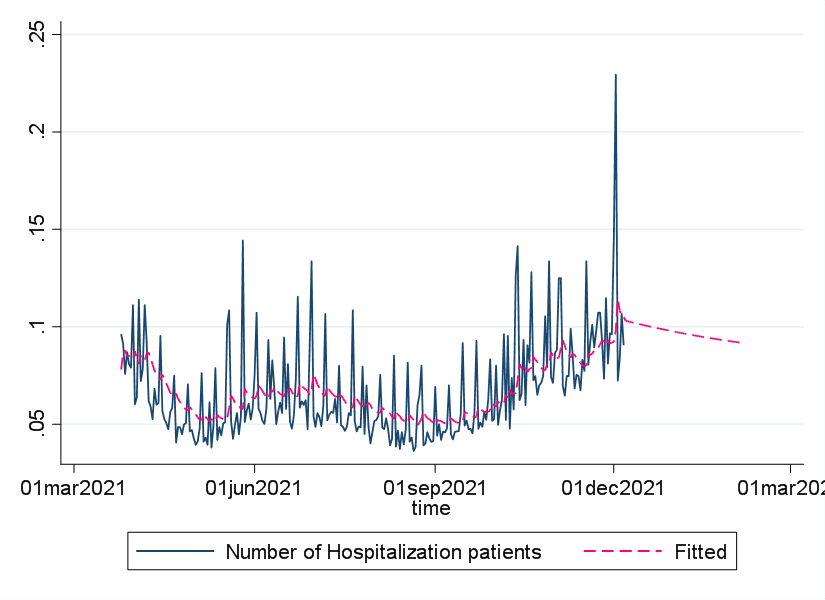** |
| --- | --- |
| **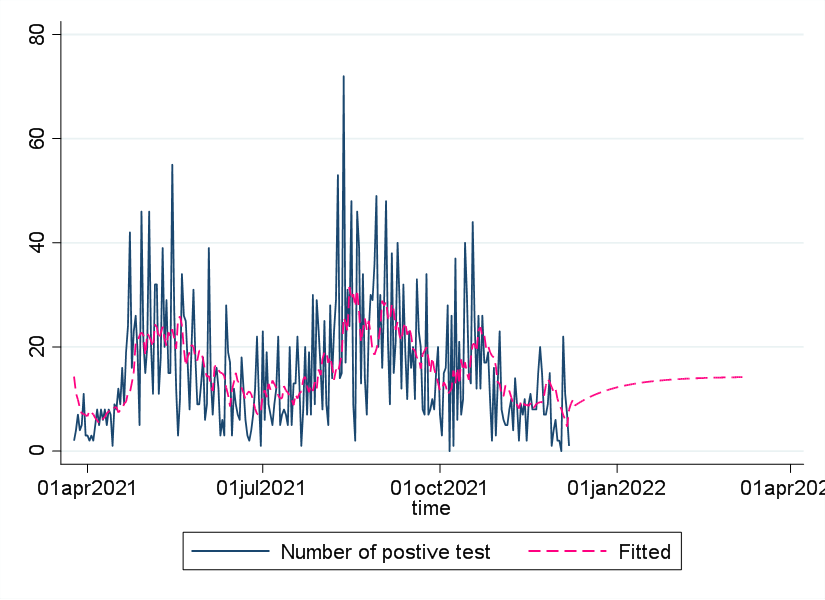** | **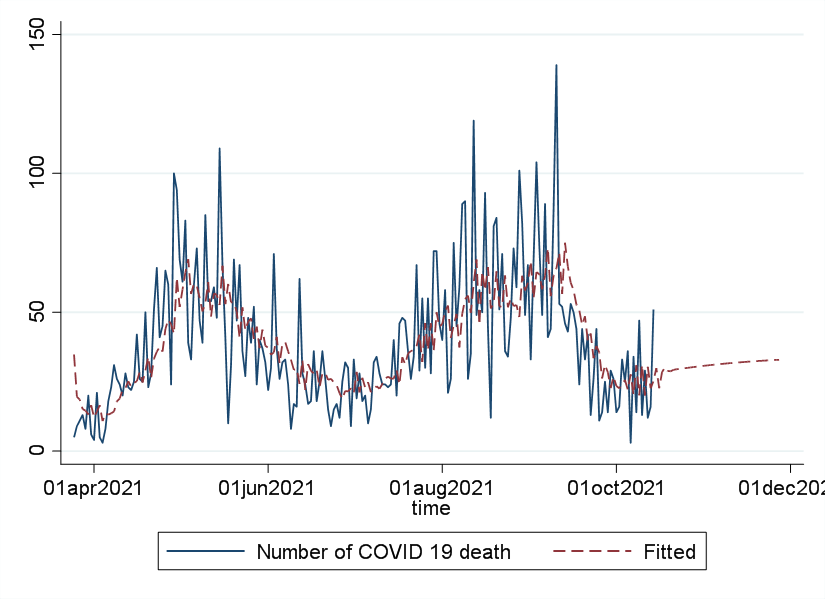** |
| **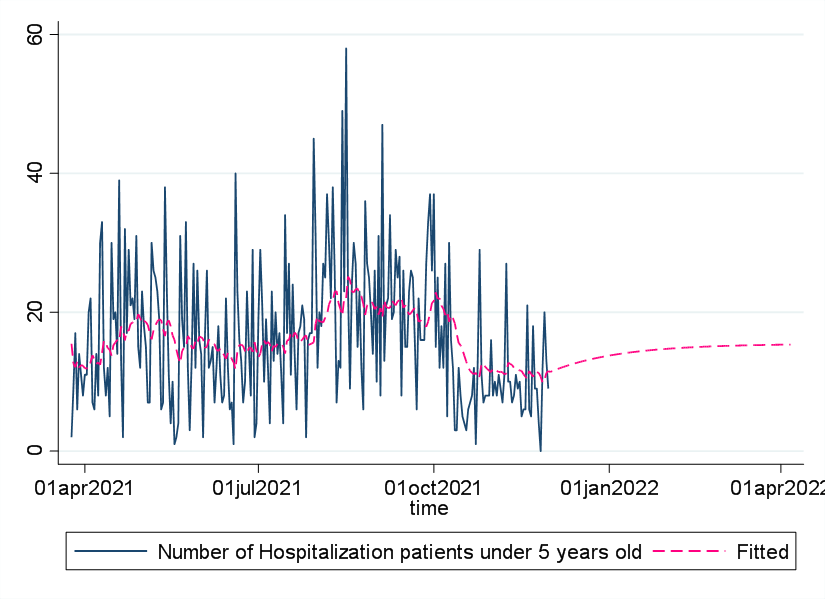** | **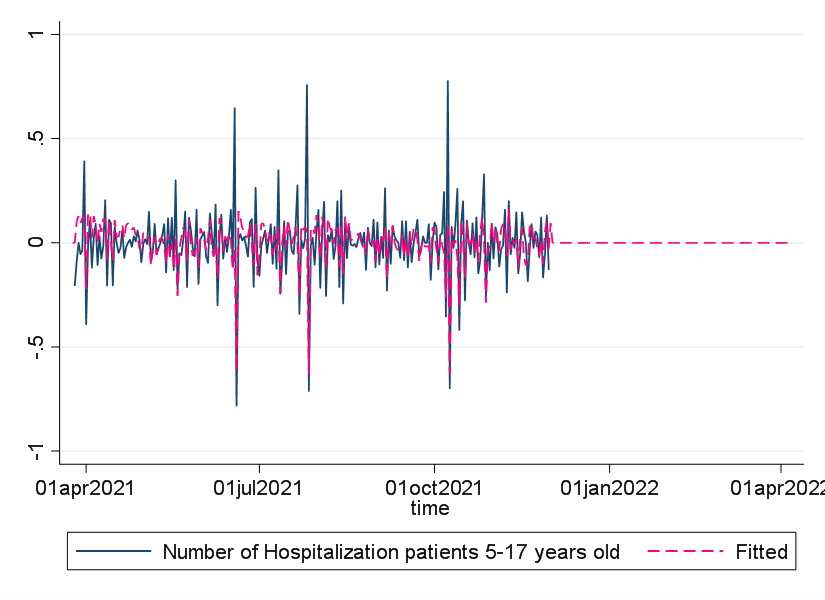** |
|  | **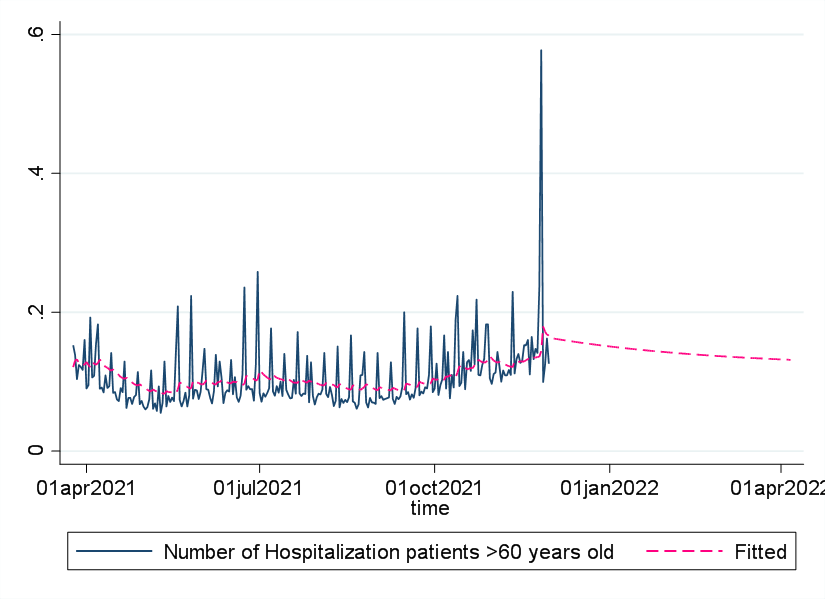** |
| **Figure S2** Time-series forecast plots for the best ARIMA models of study variables | |
